# Clinical factors are more strongly associated with prognosis than genetic alterations in early-onset colorectal cancer: a retrospective cohort study

**DOI:** 10.1186/s12885-026-16331-5

**Published:** 2026-06-11

**Authors:** Kien Trung Le, Huy Duc Tran, Minh Duc Do, Thinh Huu Nguyen, Tin Trung Nguyen, Trung Thien Tran

**Affiliations:** 1 University Medical Center HCMC, University of Medicine and Pharmacy, Ho Chi Minh City, Vietnam; 2 School of Medicine, University of Medicine and Pharmacy, Ho Chi Minh City, Vietnam

**Keywords:** Early-onset colorectal cancer, Long-term outcomes, Gene mutations, Vietnam

## Abstract

**Background:**

Early-onset colorectal cancer (EOCRC) is increasing globally and exhibits both aggressive clinicopathological features and distinct molecular characteristics. However, the relative contribution of clinical and genetic factors to prognosis remains unclear, particularly in underrepresented populations.

**Methods:**

A retrospective cohort study was conducted on 100 colorectal cancer (CRC) patients under 50 years of age, diagnosed and treated between January 2016 and December 2020, with follow-up up to 60 months or until death. Patients were stratified by patient characteristics, oncological features, and genetic mutation status to perform overall survival (OS) analysis.

**Results:**

The mean OS was 49.8 months (95% CI: 46.4–53.2), with an estimated 5-year OS rate of 65.2%. The mean disease-free survival (DFS) was 50.1 months (95% CI: 46.2–53.9). In univariate analysis, factors significantly associated with OS included serum CEA level at diagnosis (*p* = 0.001), histological grade (*p* = 0.006), mode of surgery (emergency vs. elective) (*p* = 0.003), curative resection status (*p* < 0.001), disease stage (*p* < 0.001), mutations in the POL–MMR DNA repair gene group (*p* = 0.035), high mutation accumulation (*p* = 0.020), *PIK3CA* mutation (*p* = 0.036), and co-mutation of RAS/RAF and MMR genes (*p* = 0.011). However, when adjusted for disease stage, only patient and oncological factors remained associated with OS, including elevated CEA (HR = 2.33, 95% CI: 1.2–4.7, *p* = 0.018), poorly differentiated tumors (HR = 2.57, 95% CI: 1.2–5.7, *p* = 0.021), emergency surgery (HR = 3.50, 95% CI: 1.5–7.9, *p* = 0.003), and curative treatment (HR = 0.10, 95% CI: 0.05–0.2, *p* < 0.001).

**Conclusion:**

In EOCRC, clinical and treatment-related factors remained independently associated with overall survival after adjustment for disease stage, whereas the evaluated genetic variables did not retain statistical significance. These findings highlight the importance of clinical management strategies, while the prognostic value of genetic alterations requires further investigation.

## Background

Early-onset colorectal cancer (EOCRC) has emerged as a growing clinical challenge, with a rising incidence among individuals under 50 years of age despite stable or declining rates in older populations [1]. In Vietnam, EOCRC accounts for a substantial proportion of colorectal cancer cases, ranging from 11.7% to 30% [2–4]. This trend has prompted increasing attention toward understanding the unique biological and clinical behavior of this disease.

A paradox has emerged in the current literature. On one hand, EOCRC is frequently associated with aggressive clinicopathological features, including advanced stage at diagnosis, left-sided tumor predominance, and unfavorable histology [5–8], all of which are traditionally linked to poor prognosis. On the other hand, EOCRC is also characterized by a higher prevalence of hereditary cancer syndromes and distinct molecular alterations, including mutations in DNA repair pathways and other oncogenic drivers [5, 6, 8–14], some of which have been associated with more favorable outcomes or improved treatment responsiveness.

This apparent contradiction raises an important question: to what extent do molecular alterations independently influence prognosis in EOCRC, beyond their association with disease stage and tumor characteristics? While advances in genomic profiling have fueled expectations for precision oncology, the relative contribution of genetic versus clinical factors to survival outcomes remains unclear, particularly in underrepresented populations.

To address this gap, the present study evaluates long-term survival and identifies independent prognostic factors in EOCRC, integrating both clinicopathological characteristics and genetic alterations in a Vietnamese cohort.

## Materials and methods

### Study design and patient selection

**Fig. 1:**
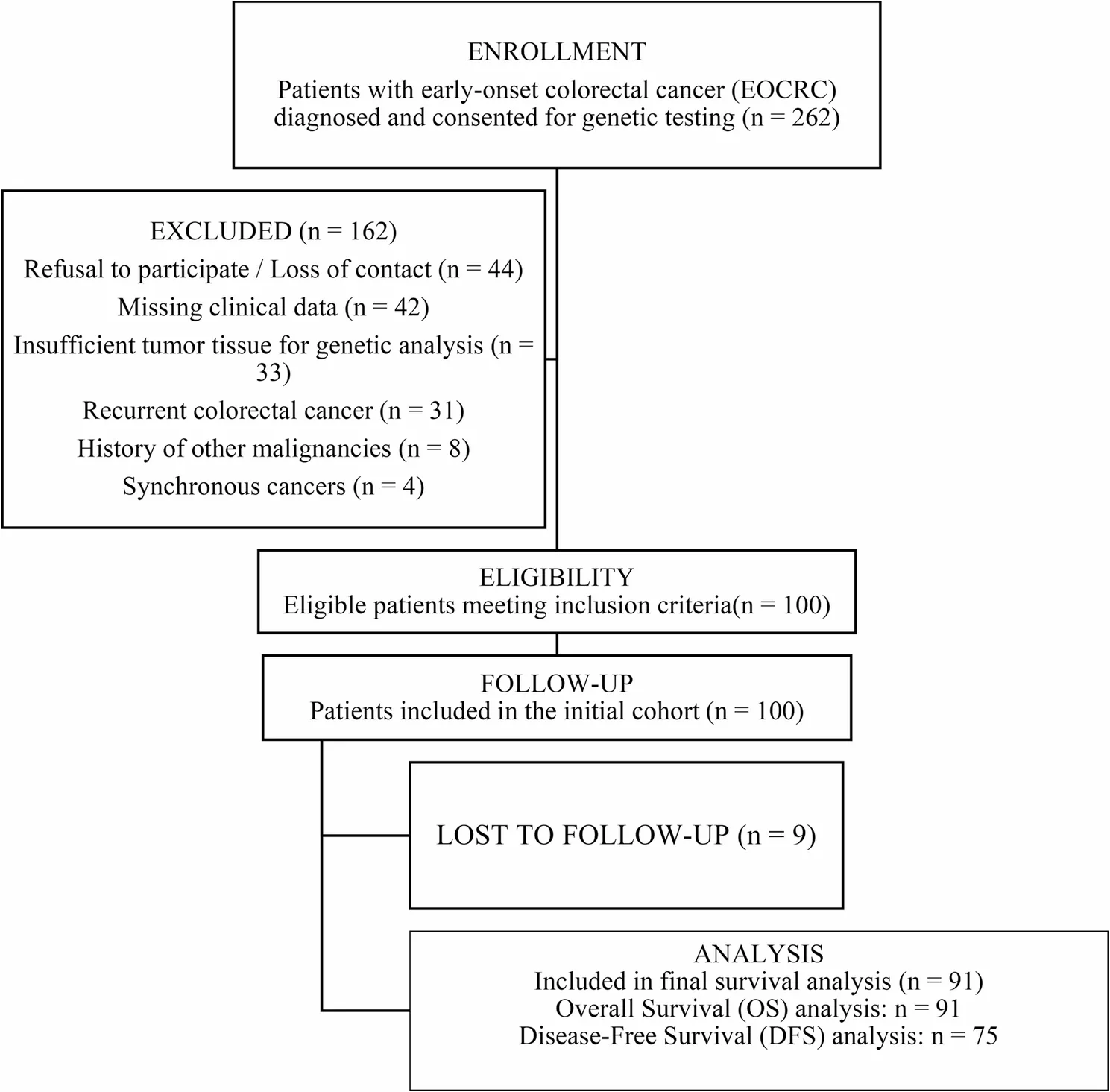
STROBE flow diagram of patient recruitment and study selection process

We conducted a retrospective cohort study of patients younger than 50 years diagnosed with CRC at the University Medical Center, Ho Chi Minh City, between January 2016 and December 2020. Inclusion required histopathological confirmation of carcinoma and availability of tumor tissue for genetic sequencing. Patients presenting with recurrent CRC, a prior history of other primary malignancies, or synchronous non-colorectal cancers diagnosed within six months of the index CRC were excluded. The study protocol was approved by the Ethics Committee of the University Medical Center, Ho Chi Minh City (Approval No. 291/IRB-UMP), and written informed consent was obtained from all patients (Fig. 1).

### Protocol for managing patients

Patients received a comprehensive clinical workup, including physical examination, laboratory testing, and appropriate imaging modalities (colonoscopy, computed tomography, magnetic resonance imaging, or positron emission tomography–computed tomography as indicated) for tumor staging according to the 8th edition of the American Joint Committee on Cancer (AJCC) [15]. Treatment plans were established through a multidisciplinary tumor board involving surgeons, radiologists, oncologists, and pathologists, in line with the guidelines of the Vietnamese Ministry of Health. Follow-up was conducted at regular intervals for up to 60 months after treatment or until death, whichever occurred first.

### Protocol for somatic mutation analysis

Tumor tissue samples from all patients were subjected to DNA extraction. Somatic alterations were assessed using a targeted sequencing panel of 21 genes, comprising PIK3CA, KRAS, NRAS, BRAF, APC, TP53, STK11, PTEN, BMPR1A, SMAD4, CHEK2, POLD1, POLE, MUTYH, MLH1, MSH2, MSH6, PMS2, EPCAM, ATM, and CDH1. The genomic analyses were based on previously published sequencing data from paired blood and tumor tissue samples of the same cohort. Detailed information regarding laboratory workflows, sequencing procedures, variant analysis and interpretation, as well as the results of next-generation sequencing and their confirmation by Sanger sequencing, has been described previously [16].

### Clinical data collection and statistical analysis

Clinical data were retrieved from institutional databases, with missing variables supplemented through follow-up when available. Baseline variables comprised demographic characteristics (age, sex, and body mass index), comorbidities, history of bowel disease, clinical presentation, hematological parameters, serum carcinoembryonic antigen (CEA) levels, serum albumin levels, tumor characteristics, initial treatment modality, TNM stage, follow-up duration, recurrence status, and CRC-related genetic alterations. .

Patients were categorized according to tumor location (right-sided colon cancer, left-sided colon cancer, and rectal cancer), age, sex, CEA level at diagnosis, histological grade and type, disease stage (early: I–II; advanced: III–IV), mode of surgery (emergency vs. elective), curative resection status, and genetic mutation status for prognostic analyses.

Mutations in KRAS and BRAF were collectively classified as RAS/RAF mutations (no NRAS mutations were identified in this study). Mutations in proofreading polymerase genes (POLE and POLD1) and mismatch repair (MMR) genes associated with Lynch syndrome (MLH1, MSH2, MSH6, and PMS2) were categorized as the POL–MMR DNA repair gene group. POLE/POLD1 and MMR gene alterations were grouped because defects in both pathways are associated with hypermutated phenotypes, increased tumor immunogenicity, and potential sensitivity to immune checkpoint inhibitors. Cases harboring mutations in more than three genes or more than three pathogenic variants among the 21 analyzed genes were defined as having a high mutation accumulation.

Survival analyses were performed using both single-gene and multi-gene models. The single-gene model included the five most frequently mutated genes (TP53, APC, KRAS, PIK3CA, and SMAD4), and BRAF was additionally included as a gene of interest despite not being among the five most frequently mutated genes. Multi-gene models included: (i) POL–MMR gene mutations; (ii) high mutation accumulation; (iii) MMR gene mutations in cases concurrently harboring RAS/RAF mutations; (iv) a co-mutation model of APC/KRAS/TP53, representing a canonical mutational pattern of CRC arising through the classical adenoma–carcinoma sequence within the chromosomal instability (CIN) pathway; and (v) a triple wild-type model involving APC, BRAF, and MMR genes, explored as a surrogate genotype that may suggest a MACS-like phenotype rather than a definitive molecular classification of the MACS subtype.

Categorical variables were analyzed using the Chi-square or Fisher’s exact test, whereas continuous variables were compared using the Independent-samples T-test, Welch’s t-test, or the nonparametric Mann–Whitney U test, as appropriate. OS was calculated from the date of diagnosis to the last follow-up or death from any cause, and DFS comprised the interval from curative surgery to tumor recurrence or death, whichever occurred first. Survival outcomes were evaluated using the Kaplan–Meier method, and differences between groups were assessed with the log-rank test. Because fewer than 50% of patients experienced the event of interest during follow-up, median survival could not be reliably estimated; therefore, mean survival times with 95% confidence intervals (CIs) were reported. Variables with *p* < 0.05 in univariate analysis were subsequently evaluated in stage-adjusted multivariable Cox regression models. Because of the limited number of outcome events, genetic variables were assessed in separate models to minimize the risk of overfitting. Prognostic factors were assessed by univariate and multivariate analyses using the Cox proportional hazards regression model. Statistical significance was defined as a p value < 0.05. All analyses were conducted using IBM SPSS Statistics (IBM Corp., Armonk, NY, USA).

## Results

Between 2016 and 2020, a total of 100 patients with EOCRC aged 18–49 years who met the inclusion criteria were enrolled. Patients with left-side colon cancer and rectal cancer had significantly higher mean serum CEA levels at diagnosis (*p* = 0.023), whereas right-sided colon cancer was more frequently associated with mucinous or signet-ring cell histology (*p* = 0.001). The overall rate of curative resection was 84%, including all patients with stage I–II disease (100%) and 98% of those with stage III disease; in contrast, only four patients with stage IV disease (21.1%) underwent curative resection. Emergency surgery was required in 15% of cases. The mean OS was 49.8 months (95% CI: 46.4–53.2), with an estimated 5-year OS rate of 65.2 (Table 1).

**Table 1:** Clinicopathological characteristics and long-term outcomes of the patients

Factors	Total (*n* = 100)	Right colon cancer (*n* = 31)	Left colon cancer (*n* = 26)	Rectal cancer (*n* = 43)	*p* value
Age, mean	37.4	37.9	34.7	38.7	0.074
Sex, n (%)					0.442
Male	52	19 (61.3%)	13 (50%)	20 (46.5%)	
Female	48	12 (38.7%)	13 (50%)	23 (53.5%)	
CEA					0.169
< 5 ng/mL	70	23 (74,2%)	21 (80,8%)	26 (60,5%)	
≥ 5 ng/mL	30	8 (25,8%)	5 (19,2%)	17 (39,5%)	
Mean value (ng/mL)	47.6	3.8	121.3	34.6	**0.023**
Histological grade, n (%)					0.265
Well and moderately differentiated	87	25 (80.6%)	22 (84.6%)	40 (93%)	
Poorly differentiated	13	6 (19.4%)	4 (15.4%)	3 (7%)	
Histological type, n (%)					
Adenocarcinoma	88	22 (71%)	24 (92.3%)	42 (97.7%)	**0.001**
Mucinous or signet ring cell carcinoma	12	9 (29%)	2 (7.7%)	1 (2.3%)	
Disease stage, n (%)					
I + II	32	13 (41.9%)	9 (34.6%)	10 (23.3%)	0.223
III + IV	68	18 (58.1%)	17 (65.4%)	33 (76.7%)	
OS mean (month)	49.8	48.5	46.3	52.5	0.862
5-year survival rates (%)	65.2	63.8	64.1	66.3	
DFS mean (month)	50.1	53	45.6	51	0.703

Overall, somatic gene mutations were detected in 96% of cases, with the most frequently altered genes being TP53 (66%), APC (52%), KRAS (43%), PIK3CA (27%), and SMAD4 (18%). No mutations were identified in NRAS, STK11, or EPCAM. Mutations in the POL–MMR DNA repair gene group were observed in 30% of patients, including MMR gene alterations in 25% and POL gene alterations in 7%.

**Fig. 2:**
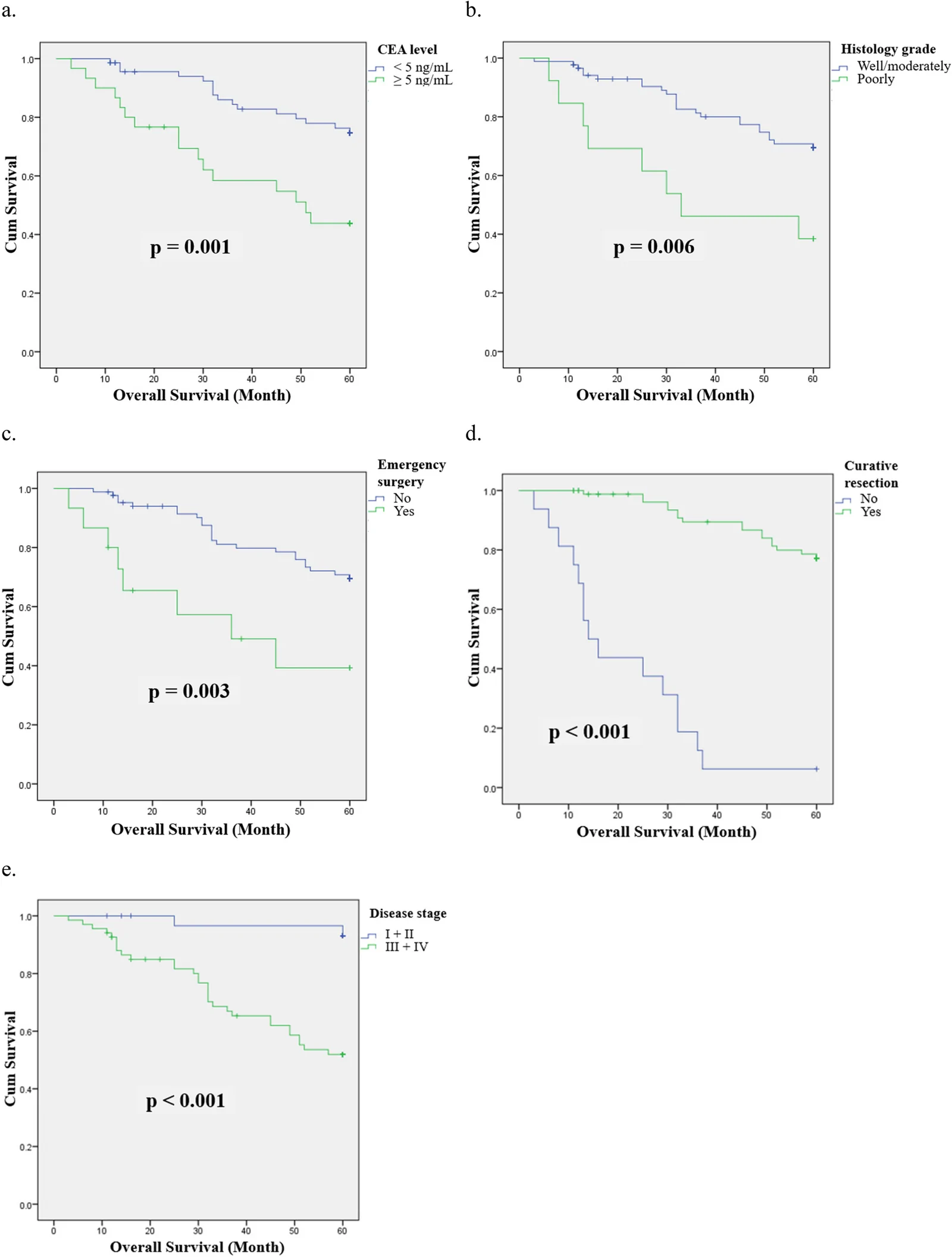
OS stratified by **a** CEA level, **b** histological grade, **c **emergency surgery, **d** curative resection, and **e** disease stage

In univariate analysis, factors significantly associated with OS included serum CEA level at diagnosis, histological grade, mode of surgery (emergency vs. elective), curative resection status, disease stage, mutations in the POL–MMR DNA repair gene group, high mutation accumulation, PIK3CA mutation, and co-mutation of RAS/RAF and MMR genes. Specifically, patients with elevated CEA had a shorter OS than those with normal CEA (41.0 vs. 53.7 months, *p* = 0.001); poorly differentiated tumors were associated with worse OS compared with well/moderately differentiated tumors (37.4 vs. 51.7 months, *p* = 0.006). Patients undergoing emergency surgery had inferior OS compared with those receiving elective surgery (36.3 vs. 52.1 months, *p* = 0.003), and curative resection was associated with markedly better survival than non-curative treatment (55.5 vs. 21.7 months, *p* < 0.001). Advanced-stage disease (III–IV) was associated with shorter OS compared with early-stage disease (I–II) (45.6 vs. 58.8 months, *p* < 0.001) (Fig. 2).

**Fig. 3:**
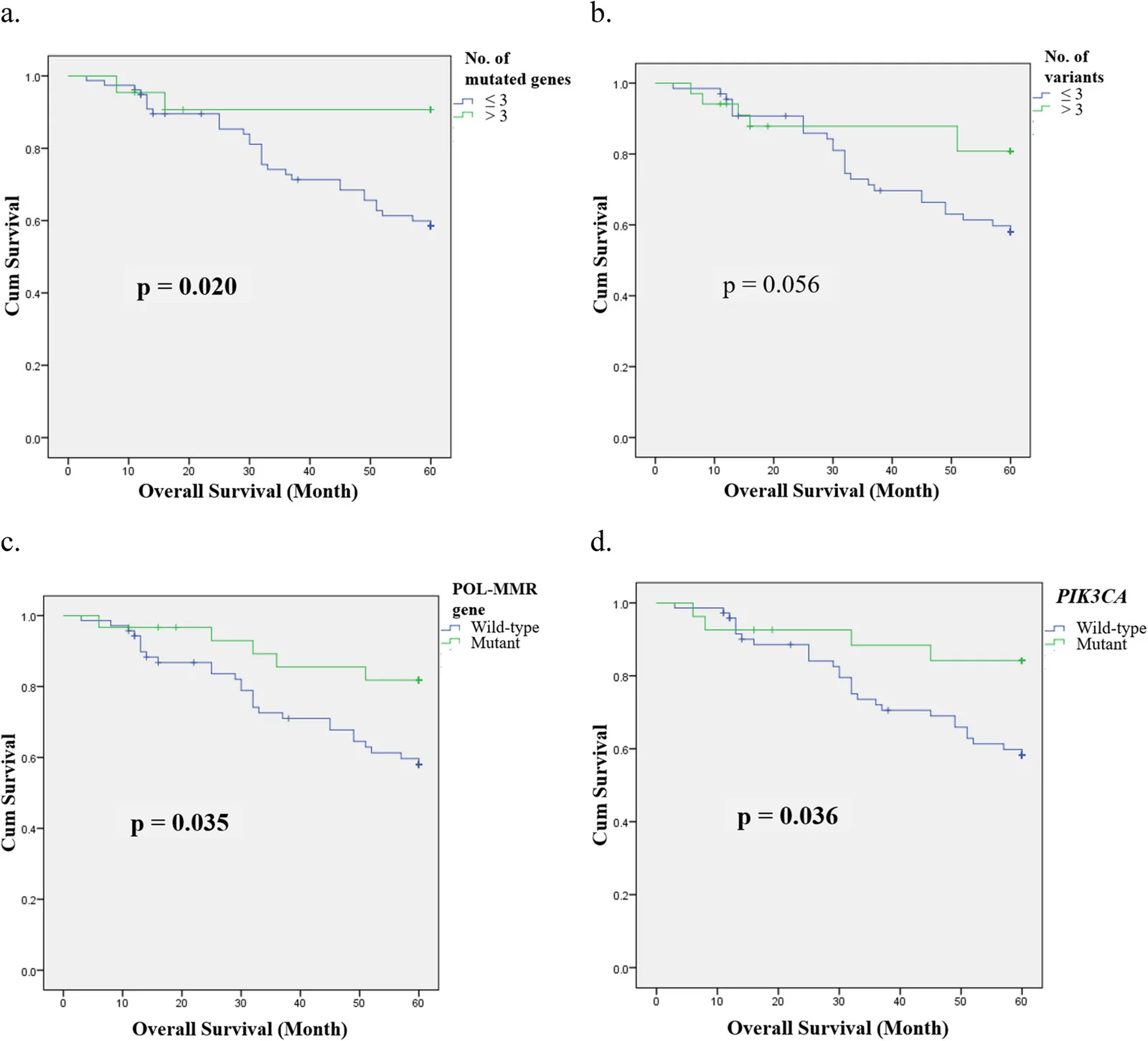
OS stratified by **a **mutation accumulation (number of mutated genes), **b** mutation accumulation (number of pathogenic variants), **c** POL–MMR mutation status, and **d** PIK3CA mutation status

We observed a strong association between POL–MMR mutations and increased mutation accumulation (number of mutated genes and number of pathogenic variants per case). Patients harboring POL–MMR mutations (54.6 vs. 47.8 months, *p* = 0.035) or exhibiting high mutation accumulation also showed better OS (55.5 vs. 48.3 months, *p* = 0.020). *PIK3CA* mutations were associated with a favorable prognostic impact (54.0 vs. 48.0 months, *p* = 0.036); however, this effect was mainly driven by exon 9 mutations (OS: 58.3 months), whereas exon 20 mutations were associated with poorer survival (OS: 44.9 months) (Fig. 3).

**Fig. 4:**
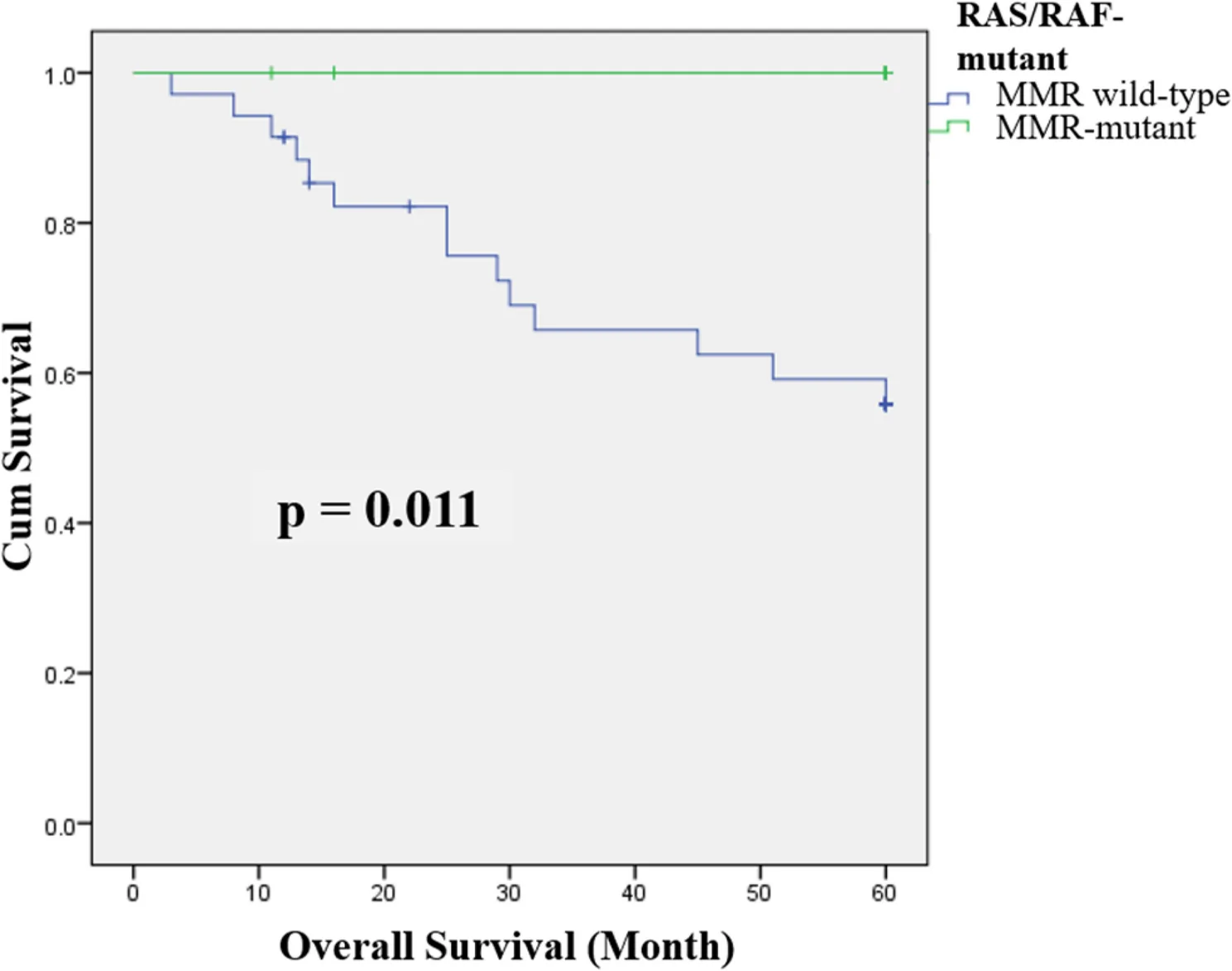
OS of patients with RAS/RAF mutations stratified by MMR mutation status

In our study, mutations in APC, TP53, KRAS, SMAD4 and BRAF were not significantly associated with prognosis. In exploratory analyses, among APC-mutated cases, the 5-year OS rate was 70.1% in patients with truncating (driver) mutations, compared with 33.3% in those with missense (passenger) mutations (p = 0.113). The overall 5-year OS rate in patients with TP53 mutations was 59.6%; specifically, carriers of the TP53 R273H variant had a 5-year OS of 50%, compared with 75.3% in TP53 wild-type patients (p = 0.165). Patients with the BRAF V600E variant had a 5-year OS rate of 50%, compared with 65.8% in those without this mutation (p = 0.397). The KRAS G12C variant was observed in cases with a high mortality rate of 66.7% in our cohort. Among patients with RAS/RAF mutations, the coexistence of MMR gene mutations was associated with a 5-year OS rate of 100%, compared with 55.9% in those without concomitant MMR mutations (p = 0.011) (Fig. 4 and Table 2).

**Table 2:** Multivariate analysis of prognostic factors stratified by disease stage

	Univariate analysis			Multivariate Analysis		
	HR	95% CI	*p*	HR	95% CI	*p*
CEA level						
< 5 ng/mL	1			1		
≥ 5 ng/mL	3.04	1.5–6.1	**0.002**	2.33	1.2–4.7	**0.018**
Histological grade						
Well/moderately differentiated	1			1		
Poorly differentiated	2.89	1.3–6.4	**0.010**	2.57	1.2–5.7	**0.021**
Emergency surgery						
No	1			1		
Yes	3.17	1.4–7.1	**0.005**	3.50	1.5–7.9	**0.003**
Curative treatment						
No	1		1			
Yes	0.07	0.03–0.1	**0.000**	0.10	0.05–0.2	**< 0.001**
Number of mutated genes						
≤ 3 genes	1		1			
> 3 genes	0.22		**0**,**035**	0.27	0.07–1.1	0,076
POL–MMR gene group						
No	1			1		
Yes	0.38	0.14–0.97	**0**,**044**	0.44	0.17–1.1	0,092
PIK3CA mutation						
No	1			1		
Yes	0.34	0.12–0.99	**0**,**047**	0.49	0.17–1.4	0,180
RAS/RAF mutation*						
Without MMR mutation	1			1		
With MMR mutation	-	-	-	-	-	-

*Because no death events were observed in patients with concurrent KRAS/BRAF and MMR mutations, this variable was not eligible for inclusion in the multivariate analysis

However, in stage-stratified multivariate analysis, variables related to genetic mutations were no longer significantly associated with overall survival, in contrast to the remaining clinical and pathological factors.

## Discussion

In our study, disease stage, baseline serum CEA level, tumor differentiation, surgical setting (emergency vs. elective), and the ability to achieve curative resection were identified as independent prognostic factors after adjustment. Consistent with previous reports, serum CEA remains a robust biomarker for prognosis in colorectal cancer. Elevated pre-treatment CEA has been associated with higher recurrence rates and significantly worse survival outcomes across multiple cohorts [17–21], including reduced OS and DFS, poorer tumor response, and inferior regression after treatment.

Tumor differentiation was also an important determinant of outcome. Previous studies have shown that poor differentiation is associated with adverse survival, particularly in advanced disease, and correlates with aggressive pathological features such as deeper invasion, lymph node involvement, and lymphovascular invasion [1, 22, 23]. Notably, poorly differentiated tumors may confer worse outcomes even in early-stage disease [22], while specific histological subtypes such as signet ring cell carcinoma have also been identified as independent predictors of reduced survival [24].

Surgical factors played a critical role in prognosis. Patients undergoing emergency surgery or those not eligible for curative resection had markedly poorer outcomes, largely reflecting more advanced disease at presentation. Emergency conditions such as obstruction or perforation are associated with higher perioperative morbidity, suboptimal oncological resection, and limited access to adjuvant therapy, all of which contribute to inferior survival [25–27].

Although genetic alterations were associated with overall survival in univariate analysis, they did not retain independent significance in multivariable models. This finding suggests that their apparent prognostic impact may be mediated by their association with tumor stage and biological behavior. Nevertheless, our results are consistent with prior evidence indicating that tumors harboring POL–MMR mutations or high mutation burden may have more favorable outcomes, likely due to increased tumor immunogenicity associated with tumor mutational burden (TMB) or microsatellite instability (MSI) [11, 28–32]. Although TMB was not directly quantified and MSI status was not comprehensively characterized in the present study, previous studies have suggested that tumors with defective DNA repair mechanisms may exhibit biological features associated with MSI-H status or elevated TMB. Improved survival in dMMR/MSI-H tumors has also been attributed to enhanced immune infiltration and better responsiveness to systemic therapy [29, 31, 33].

The prognostic role of PIK3CA mutations remains heterogeneous and context-dependent [34]. In our cohort, exon 9 mutations were associated with improved survival, whereas exon 20 mutations were linked to poorer outcomes, in line with previous reports [29–31, 33, 34]. In addition, concurrent MMR mutations appeared to attenuate the adverse prognostic impact of RAS/RAF alterations, supporting the hypothesis that MSI status may modify their biological effects [28, 30].

Recent large-scale molecular studie have further demonstrated that EOCRC represents a biologically heterogeneous disease with distinct genomic and clinicopathological characteristics compared with late-onset CRC [35]. This study provides a comprehensive evaluation of both clinical and molecular determinants of prognosis in EOCRC within a real-world Vietnamese cohort, a population that remains underrepresented in the literature. The integration of clinicopathological data with a targeted 21-gene mutation panel, together with multivariable analysis, strengthens the validity of our findings and clarifies the relative contributions of clinical and genetic factors. From a clinical perspective, our results highlight that optimizing management strategies—including early detection, appropriate surgical timing, and achieving curative resection—remains the most critical approach to improving outcomes. While molecular profiling offers valuable biological insights and may inform targeted or immunotherapy, its prognostic role appears to be context-dependent and should be interpreted alongside established clinical parameters.

This study has several limitations. The relatively small sample size and limited number of outcome and recurrence events may have reduced statistical power, particularly for analyses involving less frequent genetic alterations and DFS analyses, and may have affected the stability of some estimates. Several genetic analyses were exploratory in nature and should be interpreted with caution pending validation in independent cohorts. The retrospective, single-center design may introduce selection bias and limit generalizability. In addition, the lack of a comparison group of late-onset colorectal cancer patients precludes direct evaluation of age-related differences in prognostic factors. Finally, the gene panel used, although comprehensive, does not capture the full spectrum of genomic alterations.

## Conclusion

Baseline serum CEA level, tumor differentiation, surgical setting (emergency vs. elective), and the ability to achieve curative resection were independent prognostic factors in early-onset colorectal cancer after adjustment for disease stage. In contrast, genetic alterations—including POL–MMR mutations, high mutation accumulation, PIK3CA mutations, and MMR alterations in tumors harboring RAS/RAF mutations—were associated with survival only in univariate analysis and did not retain independent significance in multivariable models.

## Data Availability

The dataset generated and/or analyzed during the current study is publicly available in the Mendeley Data repository at: https://doi.org/10.17632/wzb623w2sz.1.

## Abbreviations

CRC: Colorectal cancer
EOCRC: Early-onset colorectal cancer
LOCRC: Late-onset colorectal cancer
OS: Overall survival
DFS: Disease-free survival
CEA: Carcinoembryonic antigen
MMR: Mismatch repair
dMMR: Deficient mismatch repair
pMMR: Proficient mismatch repair
MSI: Microsatellite instability
MSI-H: Microsatellite instability–high
MSS: Microsatellite stable
TMB: Tumor mutational burden
POL: DNA polymerase proofreading genes (POLE/POLD1)
RAS: Rat sarcoma viral oncogene
RAF: Rapidly accelerated fibrosarcoma
PIK3CA: Phosphatidylinositol-4,5-bisphosphate 3-kinase catalytic subunit alpha
NGS: Next-generation sequencing
HR: Hazard ratio
CI: Confidence interval
IRB: Institutional review board
